# Intranasal Delivery of E-Selectin Reduces Atherosclerosis in ApoE−/− Mice

**DOI:** 10.1371/journal.pone.0020620

**Published:** 2011-06-20

**Authors:** Xinhui Li, Kory R. Johnson, Mark Bryant, Abdel G. Elkahloun, Marcelo Amar, Alan T. Remaley, Ranil De Silva, John M. Hallenbeck, Jacqueline A. Quandt

**Affiliations:** 1 Stroke Branch, National Institute of Neurological Disorders and Stroke, National Institutes of Health, Bethesda, Maryland, United States of America; 2 Bioinformatics Section, National Institute of Neurological Disorders and Stroke, National Institutes of Health, Bethesda, Maryland, United States of America; 3 Division of Veterinary Resources, Office of Research Support, National Institutes of Health, Bethesda, Maryland, United States of America; 4 Division of Intramural Research Programs Microarray Core Facility, National Institutes of Health, Bethesda, Maryland, United States of America; 5 National Heart, Lung and Blood Institute, National Institutes of Health, Bethesda, Maryland, United States of America; 6 Neuroimmunology Branch, National Institute of Neurological Disorders and Stroke, National Institutes of Health, Bethesda, Maryland, United States of America; Universität Würzburg, Germany

## Abstract

Mucosal tolerance to E-selectin prevents stroke and protects against ischemic brain damage in experimental models of stroke studying healthy animals or spontaneously hypertensive stroke-prone rats. A reduction in inflammation and neural damage was associated with immunomodulatory or “tolerogenic” responses to E-selectin. The purpose of the current study on ApoE deficient mice is to assess the capacity of this stroke prevention innovation to influence atherosclerosis, a major underlying cause for ischemic strokes; human E-selectin is being translated as a potential clinical prevention strategy for secondary stroke. Female ApoE**−/−** mice received intranasal delivery of E-selectin prior to (pre-tolerization) or simultaneously with initiation of a high-fat diet. After 7 weeks on the high-fat diet, lipid lesions in the aorta, serum triglycerides, and total cholesterol were assessed as markers of atherosclerosis development. We also assessed E-selectin-specific antibodies and cytokine responses, in addition to inflammatory responses that included macrophage infiltration of the aorta and altered gene expression profiles of aortic mRNA. Intranasal delivery of E-selectin prior to initiation of high-fat chow decreased atherosclerosis, serum total cholesterol, and expression of the leucocyte chemoattractant CCL21 that is typically upregulated in atherosclerotic lesions of ApoE**−/−** mice. This response was associated with the induction of E-selectin specific cells producing the immunomodulatory cytokine IL-10 and immunosuppressive antibody isotypes. Intranasal administration of E-selectin generates E-selectin specific immune responses that are immunosuppressive in nature and can ameliorate atherosclerosis, a major risk factor for ischemic stroke. These results provide additional preclinical support for the potential of induction of mucosal tolerance to E-selectin to prevent stroke.

## Introduction

Atherosclerosis is a major cause and contributor to stroke. With 15 million strokes each year that result in death or permanent disability in 2/3 of cases, stroke is the second leading cause of death and the leading cause of long-term disability worldwide [1]. This burden will increase greatly during the next two decades due to population aging [2]. Despite decades of intensive research, therapeutic options for acute stroke are still very limited and only a small proportion of patients can benefit from recombinant tissue plasminogen activator, aspirin, or hemispheric decompression [2]. In this regard, alleviating factors contributing to stroke as a preventative measure is critical for lowering stroke-associated mortality or disability. Additionally, it cannot be ignored that other organs (e.g. heart, kidney) and multiple vascular beds are also affected by atherosclerosis [3], [4].

From atherosclerotic plaque development within arterial walls to end-stage thrombotic complications, inflammatory processes play key roles in all stages of atherosclerosis [4], [5]. Limiting immune cell activation and recruitment has been shown to attenuate atherosclerosis and its complications [6], [7]. In several diseases with inflammatory or autoimmune components, T cells with immunosuppressive qualities, including T regulatory cells (Tregs), are indispensable for maintaining immune homeostasis and unresponsiveness to self-antigen [8]. Mechanisms of Treg suppression vary depending on the type of cell, but can involve cell-contact dependent or independent pathways, suppressor cytokines (TGF-β, IL-10, etc.), and immune cell killing [9]. In the model of mucosal tolerance [10], it has been shown that secondary to mucosal administration of an antigen, the associated lymphoid tissues preferentially prime T helper lymphocytes that produce either IL-10 or TGF-ß upon subsequent antigen exposure. In this regard, oral or intranasal delivery of a variety of antigens to induce Tregs and associated immunomodulation has been used successfully in treating atherosclerosis, autoimmune diseases, transplant rejection, and allergy in several animal models [11], [12], [13], [14], [15], [16].

E-selectin is a cell surface glycoprotein adhesion molecule that is induced exclusively on activated endothelium and mediates the adhesion of leukocytes [17]. Cleavage of its extracellular domain leads to the detection of soluble E-selectin in serum, increased levels of which have been observed in subarachnoid hemorrhage [18], atherosclerosis [19], multiple sclerosis [20], end stage renal disease [21], and acute strokes [22]. Our group has found that intranasal delivery of E-selectin prevents spontaneous ischemic and hemorrhagic strokes [23] and protects against ischemic brain damage [24]. Intranasal delivery of E-selectin has been shown to influence many inflammatory and neuroprotective aspects of cerebrovascular disease, including the ability to increase the number of Treg/Foxp3+ cells in the ischemic brains [25], reduce vessel activation [26] and infiltration of CD8+ cells [24], and enhance survival of neural progenitor cells and neurons [25]. These studies employed animal models of stroke including animals with hypertension as well as healthy animals undergoing surgically-managed brain ischemia by middle cerebral artery occlusion and demonstrated the specificity to E-selectin versus irrelevant antigens such as ovalbumin (OVA) [23]. The effect of E-selectin tolerization on atherosclerosis, the underlying disease for the majority of strokes, has not been investigated.

In this study, we sought to investigate the role of intranasal delivery of E-selectin in modulating inflammatory events and lesion development in a mouse model of atherosclerosis in order to assess the capacity of this stroke prevention innovation to favorably affect this recognized stroke risk factor.

## Materials and Methods

### Ethics Statement

The study was approved by the National Institute of Neurological Disorders and Stroke Animal Care and Use Committee (Protocol #1260-06).

### Delayed type hypersensitivity (DTH) reaction

For delayed type hypersensitivity (DTH) studies, female C57BL/6 mice (Jackson Laboratory, Bar Harbor, ME) maintained on regular mouse chow underwent tolerization with either 5 µg OVA (Calbiochem, La Jolla, CA) or 5 µg human E-selectin (Novavax, Rockville, MD) at 9 weeks of age. Intranasal delivery was carried out with the animals under brief anesthesia with 1.5% isoflurane. Ten µl OVA or E-selectin (0.5 µg/µl) was delivered into one nostril every other day for 9 days (total of 5 administrations), defined as a single-round tolerization. Two weeks after the last intranasal delivery, animals were sensitized with 55 µg E-selectin in 0.5 mg/ml complete Freund's adjuvant (Difco Laboratories, Detroit, MI). Thirteen days later, animals were divided into two groups and challenged in the right and left ears with 10 µl PBS or PBS containing 5 µg E-selectin respectively. Ear swelling was measured as a relative change in ear thickness using microcalipers at 48 hrs.

### Animals and diet in atherosclerosis model

Female ApoE**−/−** mice on a C57BL/6 background (Jackson Laboratory, Bar Harbor, ME) were assigned randomly to two treatment groups receiving either human E-selectin or PBS intranasally (Biowhittaker, Walkersville, MD). Ten µl PBS or E-selectin (0.5 µg/µl) was delivered in one round of tolerization; 2 weeks later, mice received another round of tolerization. The animals were fed regular mouse chow before the start of a high-fat diet, Cocoa Butter Diet and Purina Mouse Chow (Harlan Teklad, Madison, WI). Animals were observed daily and weighed weekly.

**Figure 1:**
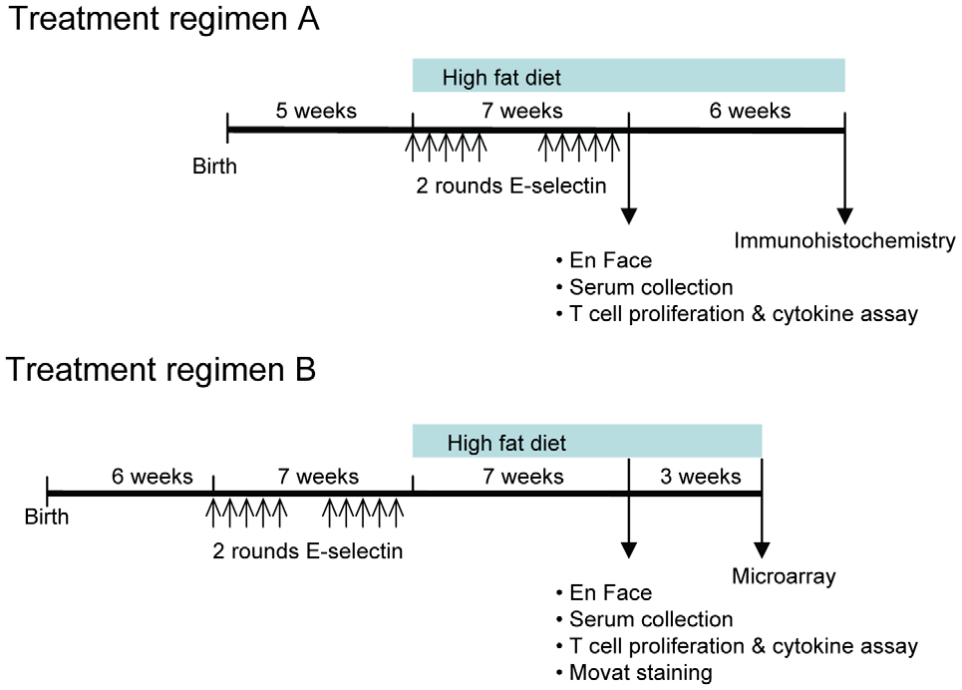
Treatment regimens. Mice received 2 rounds of intranasal delivery of E-selectin or PBS. The 2 rounds of intranasal delivery were separated by 2 weeks and each round of intranasal delivery was composed of 5 doses (PBS or 5 µg E-selectin per dose) with 1 dose (10 µl) every other day. In regimen A (N = 10 per group), we initiated the intranasal delivery of solutions and the high-fat diet simultaneously. In regimen B (N = 25 per group), we gave the mice intranasal delivery of solutions prior to initiating the high-fat diet.

Animals in this pilot study were assessed after 7 weeks on high-fat diet and having received either regimen A (N = 10 per group), with intranasal administration begun at the start of high-fat diet; or regimen B (pre-tolerization, N = 25 per group), with intranasal administrations completed just prior to the initiation of high-fat diet (Figure 1). In regimen A, after 7 weeks on high-fat diet, we collected aortas (for En Face), and splenocytes (for T cell proliferation and cytokine assays); after 13 weeks on high-fat diet, we collected upper hearts and proximal aortas for immunohistochemistry from 5 mice per group. In regimen B, after 7 weeks on a high-fat diet, we collected aortas (10 mice per group for En Face and 5 mice per group for Movet staining), and splenocytes (5 mice per group for T cell proliferation and cytokine assays); after 10 weeks on a high-fat diet, we collected aortic RNA and performed microarray (3 mice for PBS group, 4 mice for E-selectin group). For both regimens, we collected serum for all analyses performed on serum after 7 weeks on a high-fat diet (5 mice per group in regimen A and 24 mice for the PBS group and 23 mice for the E-selectin group in regimen B).

### Aortic lipid lesion quantitation and histopathology

Animals were fasted for four hours before blood draw and serum collection with Z-gel from Sarstedt (Newton, NC) according to manufacturer's instructions and stored at –80°C. After 7 weeks on high-fat diet, splenocytes were collected for cell culture and mice were perfused with 10 ml 0.9% NaCl and 10 ml perfusion solution (10% formalin, 5% sucrose, 5.8 g/L EDTA). For En Face analysis, the aorta and heart were carefully removed and stained (0.5% Sudan IV, 35% ethanol and 50% acetone) for 30 min, followed by 80% ethanol 30 min, water 1 h, and stored in perfusion solution. Extraneous fatty tissue and connective tissue were carefully trimmed away and the aorta cut longitudinally and mounted on a slide. Plaque lesions in the region extending from aortic root to 6 mm distal (including ascending aorta and arch) were quantitated using a microscopic CCD-IRIS camera and MetaMorph software (Molecular Devices, Downingtown, PA). Sections of aortic root from mice on regimen B were collected at necropsy, placed in perfusion solution, routinely processed and sectioned, and stained with Movat and Alcian blue-PAS-hematoxylin for histopathological evaluation in a blinded fashion.

### Immunohistochemistry

The upper heart and proximal aorta were embedded in Tissue-Tek OCT (Sakura Finetek USA, Torrance, CA) and sectioned serially at 10 µm thickness. Beginning with the first section containing all three valves, a section was stained every 80 µm. Five mice per treatment group and five sections per mouse were used for immunohistochemistry. Sections were fixed in cold acetone for 10 min, followed by 0.5% hydrogen peroxide 10 min, 1% BSA + 10% rabbit serum 2 h, MOMA-2 antibody (ABD Serotec, Raleigh, NC) at 1 µg/ml overnight, HRP conjugated rabbit anti-rat Fcγ (Jackson ImmunoResearch, West Grove, PA) at 0.4 µg/ml 1 h. Color was developed with DAB (Vector Lab, Burlingame, CA) and nuclei counterstained with Hematoxylin QS (Vector Lab). Rat IgG was used as a control. Macrophage positive area per cross-section and fractional macrophage positive area (macrophage positive area divided by the area inside external elastic lamina) were quantified using MetaMorph software (Molecular Devices, Downingtown, PA).

### Serum total cholesterol and triglyceride measurement

Serum total cholesterol and triglyceride levels were measured using Stanbio Cholesterol LiquiColor and Stanbio Triglyceride LiquiColor kits (Stanbio Lab, Boerne, TX) according to manufacturer's recommended protocols. For triglyceride measurement, serum was used directly; for total cholesterol, serum was diluted to 1/8 with 0.9% NaCl. The concentrations of the total cholesterol and triglyceride were calculated from a standard curve.

### Antibody assays

Plates were coated overnight with 50 µl 5 ng/µl mouse E-selectin (R&D Systems, Minneapolis, MN) in PBS, blocked with 5% BSA in PBS 1 h, incubated with 1/200 diluted mouse serum 2 h. Biotin conjugated goat anti-mouse IgG, IgG1, IgG2a and streptavidin-HRP were from Jackson ImmunoResearch. Following 1 h incubation with secondary antibody, streptavidin-HRP diluted 1/1000 was added to each well and incubated for 20 min. Color was developed with TMB (3, 3′, 5, 5′ tetramethyl benzidine, Sigma, St. Louis, MO) and stopped with 2N H_2_SO_4_. The absorbance was read at 450 nm. Fifty µl 5 ng/µl OVA coated plates were used to monitor non-specific antibody binding (with serum from OVA immunized C57BL/6 mice used as a positive control).

### T cell proliferation and cytokine assays

Splenocytes (4×10^5^) were incubated in X-vivo 15 serum free media (Lonza, Walkersville, MD) with or without 20 µg/ml E-selectin or 20 µg/ml OVA. After collecting tissue culture supernatants at 72 h, 1 µCi of [^3^H]-thymidine (PerkinElmer, Waltham, MA) was added to each well for an additional 16 h, cells were harvested on a TomTec IIIM cell harvester (Hamden, CT) and incorporated radioactivity was measured on a Wallac Microbeta Trilux (PerkinElmer, Waltham, MA). Cytokine analysis was performed by sandwich ELISA using IL-2, IL-4, IL-10, TGF-β, IL-17, IFNγ, and TNFα mouse DuoSet kits (R&D Systems) per manufacturer's directions. Serum soluble mouse E-selectin, ICAM-1, VCAM-1 and TGF-β were measured with kits from R&D. Duplicate samples were assayed.

### RNA purification, microarray, and real-time RT-PCR

After ten weeks on a high-fat diet, 3 animals receiving PBS and 4 animals receiving E-selectin on regimen B (pre-tolerization) were used in the microarray analysis. After perfusion with PBS, the aorta was carefully removed, snap frozen in liquid nitrogen, and ground with a pestle and mortar on dry ice. RNA was purified by AllPrep DNA/RNA/Protein Mini Kit (Qiagen, Valencia, CA). RNA quality and quantity was ensured using the Bioanalyzer (Agilent, Santa Clara, CA) and NanoDrop (Thermo Scientific, West Palm Beach, FL) respectively. Per RNA labeling, 200 ng of total RNA was used in conjunction with the Affymetrix recommended protocol for the Affymetrix Mouse Genome ST 1.0 GeneChip (Santa Clara, CA).

“RMA Sketch” (Expression Console, Affymetrix) was used to summarize probe measurements and generate normalized gene fragment expression values for each hybridized cRNA. Quality of data was assured via sample-level inspection by Tukey box plot, covariance-based PCA scatter plot, and correlation-based Heat Map. Gene fragments not having at least one expression value greater than system noise were deemed “non-informative” and discarded. System noise was defined as the lowest expression value at which the LOWESS fit of observed CV for each gene fragment by mean expression value for each gene fragment changes from non-linear to linear. For gene fragments not discarded, expression values were floored to equal system noise if less than system noise and subject to the Welch Modified t-test for association with tolerization. Expression values for gene fragments providing an uncorrected P-value < 0.05 and an absolute fold difference of means ≥1.25 were deemed associated with tolerization. Annotation information for genes deemed associated with tolerization were obtained via IPA (Ingenuity, Redwood City, CA).

Abundance of CCL21 mRNA was determined using QuantiTect SYBR Green RT-PCR Kit (Qiagen, Valencia, CA). The primer sequences were obtained from PrimerBank (Harvard University, Cambridge, MA) with a slight modification of the CCL21 forward primer to detect both A and B variants of CCL21; CCL21 forward primer: CATCCCGGCAATCCTGTTCT; ccl21 reverse primer: GGGGCTTTGTTTCCCTGGG; GAPDH forward primer: AGGTCGGTGTGAACGGATTTG; GAPDH reverse primer: TGTAGACCATGTAGTTGAGGTCA. Ten ng total RNA was reverse transcribed for 30 minutes at 50°C. Following 15 min at 95°C, the cDNA was amplified by 40 cycles of: 94°C 15 seconds, 62°C 30 seconds, and 72°C 30 seconds. The CCL21 expression was normalized to the expression of GAPDH and the results were expressed as fold changes in mRNA expression with respect to the control animals.

### Statistical analysis

Data are expressed as mean ± standard error of the mean (SEM). Mann Whitney Rank Sum Test was used in comparing the T cell proliferation, cytokines in cell culture supernatant, and serum antibodies between the two treatment groups. Other data passed normality tests and were analyzed by two-tailed student's t-test. Differences were considered significant at P<0.05.

## Results

### Delayed type hypersensitivity

**Figure 2:**
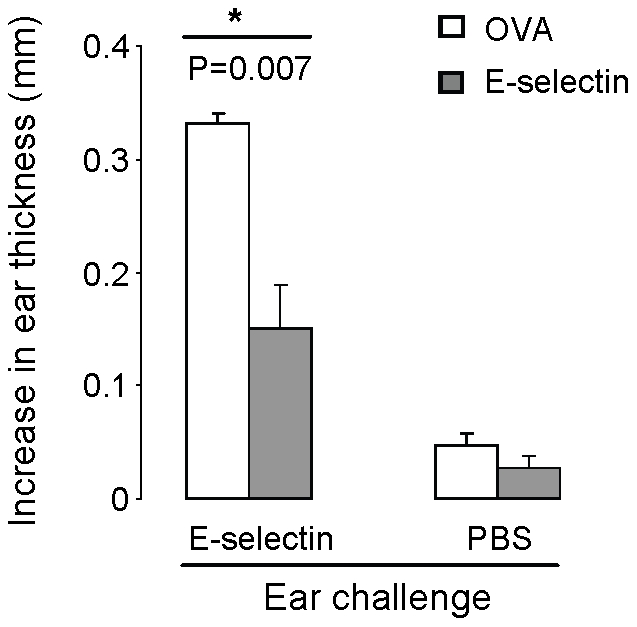
Intranasal delivery of E-selectin reduces swelling in a delayed type hypersensitivity reaction. 9 week old mice underwent one round (consisting of five intranasal administrations every other day) of 10 µl PBS containing either 5 µg OVA (white bars) or E-selectin (black bars). After sensitization and subsequent challenge with E-selectin, responses to E-selectin were significantly reduced in animals previously receiving E-selectin versus those receiving OVA intranasally (P = 0.007). Swelling was not significantly different in the ears challenged only with PBS between the two treatment groups. N = 5 mice per group, error bars represent SEM.

Previous work from our laboratory demonstrated E-selectin tolerization significantly limits Th1 mediated DTH reactions mounted in rats following sensitization and challenge with human E-selectin [23], [27], [28]. Comparisons to both PBS and OVA- tolerized animals highlighted the specificity of the response to E-selectin. After testing a range (0.1 to 10 µg) of tolerizing doses with a similar regimen in a C57BL/6 mouse model of multiple sclerosis, experimental autoimmune encephalomyelitis (EAE; Jacqueline A. Quandt, unpublished data, 2010), the 5 µg dose was found to be most effective at limiting clinical disease and was selected for the current study. In C57BL/6 mice, 5 µg of E-selectin given intranasally significantly reduced ear swelling, a response which was highly specific to E-selectin (Figure 2) with negligible reductions in OVA-tolerized mice. Intranasal E-selectin reduced swelling by more than 50% compared to OVA tolerized animals (P = 0.007) with no significant differences observed in PBS-sensitized ears. Delivery of E-selectin alone to the ear pad did not elicit swelling beyond that measured for PBS in animals naive to E-selectin (data not shown).

### Analysis of ApoE−/− animals tolerized with E-selectin

Not knowing whether E-selectin tolerization would have an effect once high-fat diet and associated atherosclerosis development were already underway, we tested the influence of E-selectin tolerization either simultaneously with initiation of a high-fat diet (regimen A) or pre-tolerization with completion prior to start of high-fat diet (regimen B). Two-rounds of intranasal delivery of 5 µg E-selectin previously afforded optimal protection in rat stroke models [24], [25] and was similarly applied in each of these regimens (Figure 1). Within each regimen, we analyzed factors associated with atherosclerosis development and additional immune properties in animals after high-fat diet consumption for 7 weeks. Daily body weight and food intake were similar between the two treatment groups, and veterinary staff monitoring animals daily noted no atypical behaviors, physical conditions nor increased incidence of lesions or infections (morbidity or mortality) in E-selectin treated animals compared to controls. Regardless of regimen, animals in the two treatment groups had similar body weight over the course of the study (Table 1).

**Table 1:** Analysis of serum lipids and cell adhesion molecules.

	PBS		E-selectin		
Characteristics	Mean ± SEM	N	Mean ± SEM	N	P
**Regimen A**					
Body weight, g	16.8 ± 0.5	5	16.7 ± 0.4	5	0.930
Triglycerides, mg/dL	NA		NA		
Total cholesterol, mg/dL	2024.3 ± 139.1	5	1912.9 ± 131.4	5	0.577
sE-selectin, ng/ml	432.1±57.8	5	425.5±47.1	5	0.931
sICAM-1, ng/ml	35.8±2.6	5	33.8±1.2	5	0.520
sVCAM-1, ng/ml	1955.2±75.7	5	2100.0±69.8	5	0.198
**Regimen B**					
Body weight, g	19.1 ± 0.3	25	19.0 ± 0.4	24	0.777
Triglycerides, mg/dL	147.7 ± 33.0	20	114.25 ± 25.5	20	0.278
**Total cholesterol, mg/dL**	**1353.7 ± 228.6**	**22**	**1143.2 ± 243.7**	**22**	**0.003**
sE-selectin, ng/ml	581.8±19.5	15	595.7±34.3	14	0.730
sICAM-1, ng/ml	38.7±1.7	21	35.2±1.5	22	0.122
sVCAM-1, ng/ml	2577.2±41.9	21	2443.2±71.5	22	0.115

Sera were collected from animals in either regimen after 7 weeks after on a high-fat diet. N indicates the number of mice.

Increased serum cholesterol is a major risk factor for the development of atherosclerosis in humans and genetically engineered mice. ApoE**−/−** mice show increased levels of serum total cholesterol and triglycerides compared to wild-type littermates that are further increased when animals receive a high-fat diet [29] and develop severe atherosclerosis. In regimen A (5 mice per group), no statistical difference in serum total cholesterol was detected between the 2 groups (Table 1) receiving PBS or E-selectin simultaneously with the introduction of high-fat diet. Animals receiving E-selectin prior to starting a high fat-diet showed modestly reduced serum total cholesterol levels (P = 0.003) compared to PBS controls, yet serum triglyceride levels were not statistically different.

### Intranasal delivery of E-selectin reduces atherosclerosis

**Figure 3:**
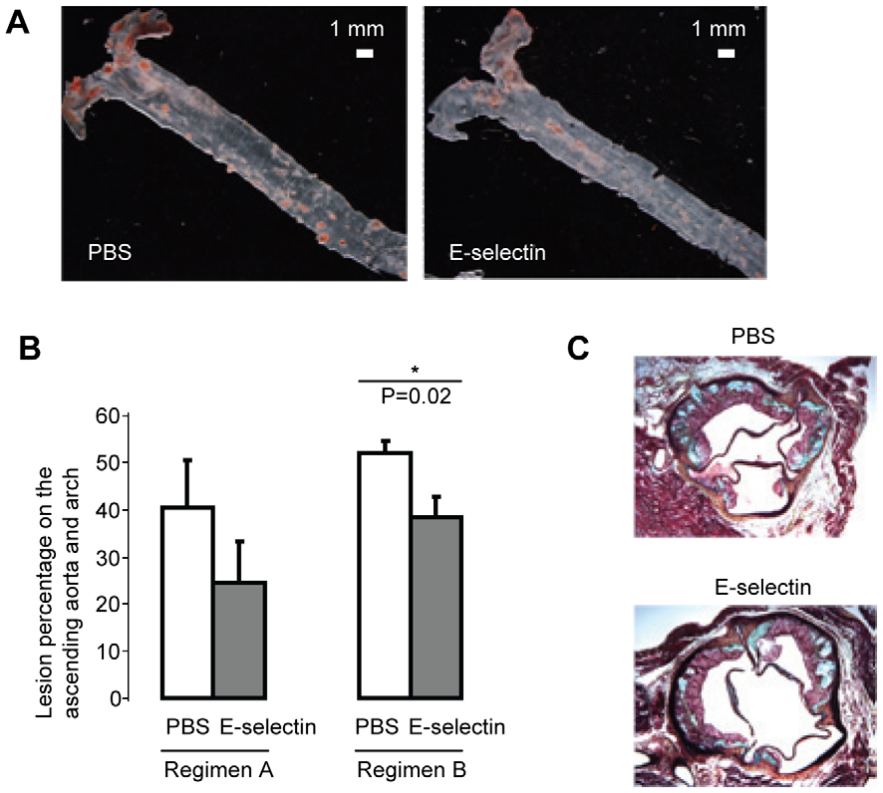
Intranasal delivery of E-selectin reduces atherosclerosis. Mice treated with regimen A (N = 5 per group) or regimen B (N = 10 per group) were processed En Face after 7 weeks on a high-fat diet. The lipid lesions in the aorta were stained with Sudan IV. (A) Representative photos illustrate the predominance of lesions in the aortic arch. (B) Quantification of lipid lesion on the ascending aorta and arch. In regimen B, pre-tolerization with E-selectin caused a significant 26.4% plaque reduction in the surface area of the ascending aorta and arch. (C) Movat staining of cross-sections of the aortic root in regimen B. The PBS and E-selectin groups showed similar degrees of pathology in the aortic roots.

We assessed atherosclerosis in En Face sections after 7 weeks on a high-fat diet. Consistent with other studies in mouse models of atherosclerosis, the majority of the lipid lesions were located near the aortic root and arch. In regimen B, 52.0±2.7% of the surface area of the ascending aorta and arch were covered with atherosclerotic lesions in PBS group, yet pre-tolerization with E-selectin caused a significant 26.4% plaque reduction (38.2±4.5% of the ascending aorta and arch, P = 0.02) (Figure 3A and B). Regimen A was less effective at reducing the atherosclerosis on the ascending aorta and arch (40.4±10.0% in PBS group versus 24.6±8.8% in E-selectin group, P = 0.27).

Where early E-selectin administration showed reduced lipid deposition, the sections of aortic roots (5 mice per group in regimen B) were stained with Movat staining (Figure 3C) and examined for endothelial damage, reticulin fiber degeneration, and basement membrane thickening or disruption. Endothelial damage was minimal in all tissues studied. Animals in both groups showed similar mild or mild to moderate reticulin fiber degeneration. Basement membranes in both groups were equally disrupted and showed similar degrees of subintimal thickening and fibrosis.

Although no difference was observed in atherosclerosis after 7 weeks in regimen A, a subset of animals receiving simultaneous E-selectin at the start of a high-fat diet were followed for an additional 6 weeks and processed to allow examination histologically. After 13-weeks on a high-fat diet, macrophage infiltration in the aortic roots of mice on regimen A was quantitated by immunohistochemistry. Mice receiving E-selectin showed a trend toward reduced macrophage infiltration compared with mice receiving PBS (4.4±0.6×10^5^ µm^2^ in PBS group versus 3.2±0.3×10^5^ µm^2^ in E-selectin group, P = 0.11). The percentage of lumen area positive for macrophages gave similar results (27.6±1.9% in PBS group versus 23.3±1.4% in E-selectin group, P = 0.11). Notably, fewer than 10 CD3^+^ or Foxp3+ cells were observed per section with no significant difference between the PBS or E-selectin treatment groups.

### Intranasal delivery of E-selectin induces E-selectin specific immune cells

**Figure 4:**
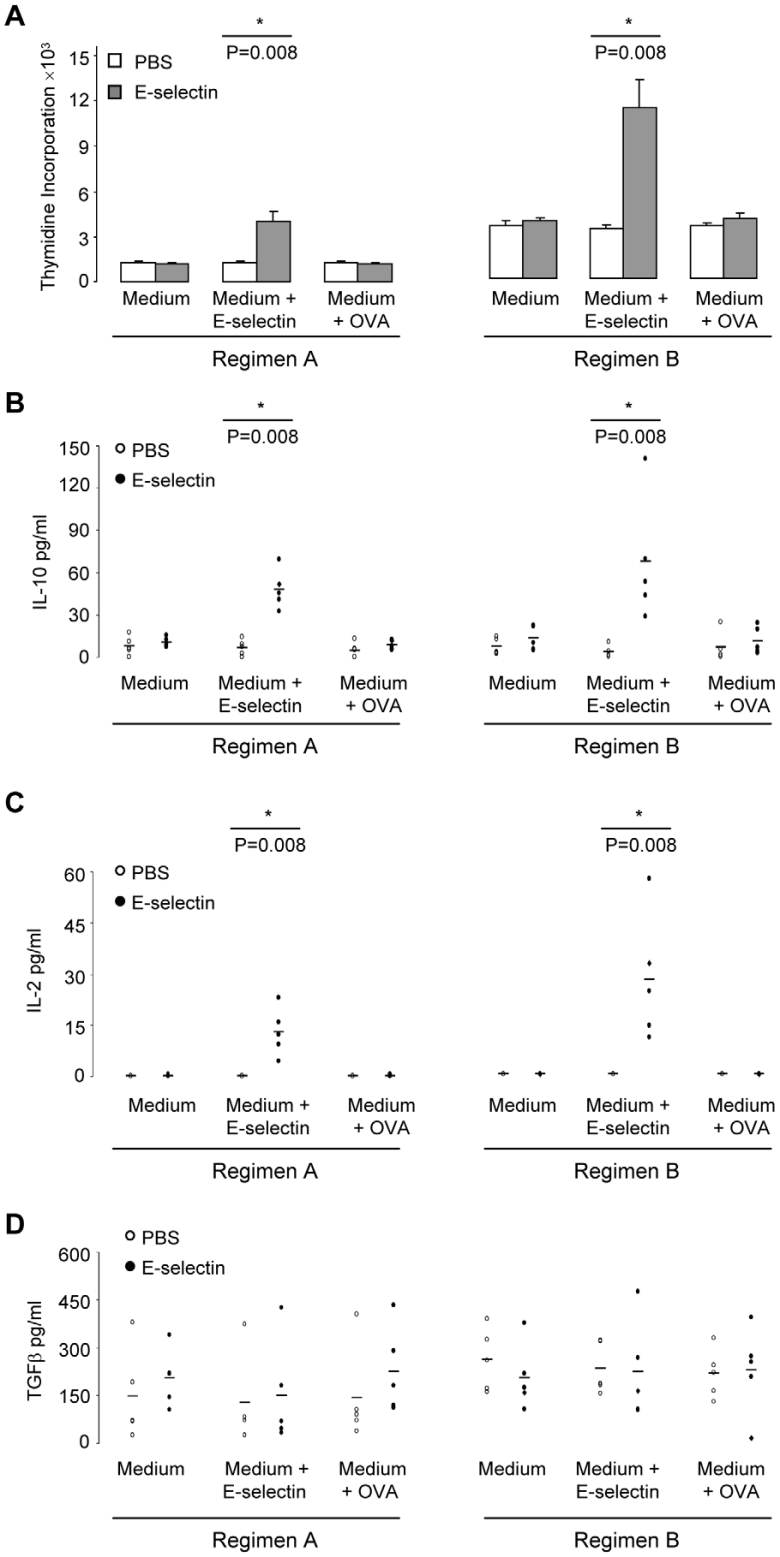
Intranasal delivery of E-selectin induces E-selectin specific regulatory cells. After 7 weeks on a high-fat diet, splenocytes were collected and cultured in X-vivo 15 alone or with 20 µg/ml E-selectin or 20 µg/ml OVA. Supernatant was collected at 72 h. Bars represent the mean of 5 animals per treatment group, error bars represent the SEM. (A) Intranasal delivery of E-selectin induces E-selectin specific immune cells. Proliferation measured by thymidine incorporation was only increased in splenocytes from animals that received intranasal delivery of E-selectin and only specifically in response to E-selectin (not the irrelevant antigen OVA). (B) Splenocytes from E-selectin tolerized mice produced more IL-10 in culture upon E-selectin stimulation. (C) Splenocytes from E-selectin tolerized mice produced more IL-2 in culture upon E-selectin stimulation. (D) Splenocytes from both treatment groups produced a similar amount of TGF-β with or without E-selectin or OVA stimulation.

Spontaneous or background proliferation of cultured splenocytes was similar for both of the PBS and E-selectin treatment groups (Figure 4A). Proliferation in response to E-selectin was observed only in animals that received E-selectin intranasally; proliferation in response to the irrelevant antigen OVA was similar to background in both groups.

Similarly, production of IL-10 and IL-2 was increased in response to E-selectin in the culture supernatant of cells from E-selectin treated mice (Figure 4B, C). The TGF-β levels in the culture supernatant were similar for both treatment groups (Figure 4D). This specificity to E-selectin is similar to data obtained in wild-type C57BL/6 mice with and without experimental autoimmune encephalomyelitis (EAE; Jacqueline A. Quandt, unpublished data, 2010). The levels of IL-4, IL-17, IFNγ and TNFα in cell culture supernatant remained below low pg/ml detection limits in both treatment regimens with or without the presence of E-selectin in culture (data not shown).

### Effects of E-selectin tolerization on mRNA expression in the aorta

Microarray analysis of mRNA was carried out in the pre-tolerization regimen B exhibiting significant alterations in lipid deposition and serum cholesterol as markers of atherosclerosis. 119/13,342 transcripts have an uncorrected P-value < 0.05 and an absolute fold-change difference ≥1.25 between the 2 treatment groups. Those gene fragments mapped to 9 known genes which were all down-regulated in the group receiving E-selectin (Table 2).

**Table 2:** Intranasal administration of E-selectin down-regulated mRNA expression of 9 genes in the aorta of ApoE**−/−** mice.

Gene Symbol	Fold change	Uncorrected P-value	Gene Description
FUT11	−2.538	0.015	Fucosyltransferase 11
DUB2A	−2.399	0.003	Deubiquitinating enzyme 2a
CCL21	−1.554	0.049	Chemokine (C-C motif) ligand 21
MACROD2	−1.513	0.018	MACRO domain containing 2
HIST1H2BG	−1.309	0.017	Histone cluster 1, H2BG
CFB	−1.286	0.021	Complement factor B
SFRP4	−1.275	0.037	Secreted frizzled-related protein 4
C4A	−1.273	0.043	Complement component 4A
APOC1	−1.255	0.021	Apolipoprotein C-I

Animals receiving PBS or E-selectin (regimen B; N = 3 and 4 respectively) were analyzed 10 weeks after a high-fat diet.

CCL21 is associated with inflammatory and vascular processes and its association to atherosclerosis has been previously defined [30]. Although CCL21 had the highest P-value (0.049) of genes identified as significant by microarray analysis, we investigated and confirmed by real-time PCR that CCL21 mRNA expression is indeed significantly down-regulated in the E-selectin group compared to PBS group (–1.75 fold, P = 0.02). Additional analysis of other genes involved in inflammation, lipid metabolism, chromatin modification and transcriptional regulation identified by microarray [31], [32] was beyond the scope of this study.

### Serum soluble E-selectin, ICAM-1, VCAM-1, and TGF-β

As a gauge of endothelial activation, we monitored serum soluble E-selectin, ICAM-1 and VCAM-1, but found no difference between the two treatment groups in either regimen (Table 1). As TGF-β is implicated in the effector function of some Treg subsets [9], we measured serum TGF-β levels in mice on regimen B. E-selectin tolerization decreased serum active TGF-ß (0.61±0.05 ng/ml in PBS group vs. 0.49±0.03 ng/ml in E-selectin group, P = 0.04, N = 23 per group). Serum total TGF-β was similar among the two groups (91.38±4.07 ng/ml in PBS group vs. 85.55±4.62 ng/ml in E-selectin group, P = 0.35, N = 23 per group). Serum IL-10, CCL21 and IFNγ were below the kit detection limits.

### Serum E-selectin specific antibodies

**Figure 5:**
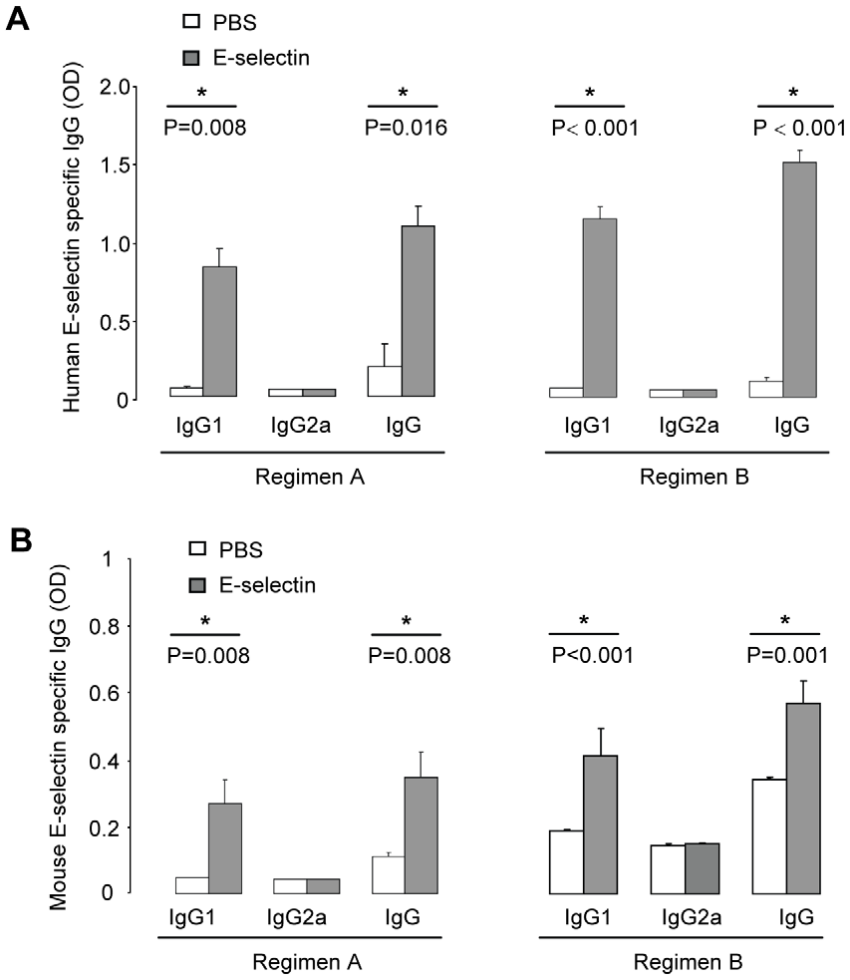
Serum E-selectin specific antibodies. Serum was collected after 7 weeks on a high-fat diet. (A) Human E-selectin specific IgG1, IgG2a and total IgG in mice on regimen A (N = 5 per group) or regimen B (N = 24 for PBS group, N = 23 for E-selectin group). (B) Mouse E-selectin specific IgG1, IgG2a and total IgG in mice on regimen A (N = 5 per group) or regimen B (N = 24 for PBS group, N = 23 for E-selectin group).

In both of the A and B regimens, serum human E-selectin-specific IgG1 and total IgG were detected in E-selectin tolerized mice (Figure 5A), whereas IgG2a levels were similarly low in both groups. The elevated serum IgG1 was proportional to elevated serum total IgG. Serum mouse E-selectin-specific IgG1 and total IgG were increased in E-selectin tolerized mice and the elevated serum IgG1 was proportional to elevated serum total IgG (Figure 5B), demonstrating cross-reactivity of human E-selectin specific antibodies to mouse E-selectin. Serum E-selectin- specific IgM was analyzed in regimen A; available serum was insufficient for this analysis in regimen B. E-selectin specific IgM did not differ between treatment groups, which is consistent with our studies in C57BL/6 mice with and without EAE (data not shown). Background antibody binding was negligible when OVA was used to coat the ELISA plates. The absorbance of serum from PBS or E-selectin treated mice was as low as the no serum control; whereas the serum levels of OVA-specific IgG and IgG1 were high in OVA immunized mice (data not shown).

## Discussion

Our previous studies in multiple preclinical disease models (stroke, vascular cognitive impairment, adult neurogenesis after brain ischemia) and the current study in atherosclerosis, have all shown that intranasal delivery of E-selectin leads to salutary mechanisms that are safe and effective. The suppression of atherogenesis after E-selectin tolerization is a new and highly desirable mechanism of action for this innovation in stroke prevention since 80% of ischemic strokes are caused by arterial occlusion secondary to atherosclerosis [33].

As we explored E-selectin tolerization in a mouse model of atherosclerosis, we tested two regimens of E-selectin administration either preceding (regimen B) or delivered simultaneously (regimen A) with a high-fat diet. The reduction of serum total cholesterol and lipid lesion on the ascending aorta and arch was statistically significant in regimen B, but not in regimen A. Regimen A may not have had sufficient power to detect the difference, yet a second marker of E-selectin efficacy, total cholesterol, also showed very little variation in this regimen and instead suggests a less effective regimen. Notably, lipid lesions on the aorta were quantitated 2 weeks after E-selectin tolerization in regimen A and 7 weeks after E-selectin tolerization in regimen B, suggesting that E-selectin tolerization may need more time to have an effect, particularly if atherosclerosis secondary to diet was already well underway. Furthermore, the mice were older in regimen B and displayed more atherosclerosis, suggesting the effect of E-selectin tolerization may be more robust or at a minimum easier to detect at a more advanced stage of disease.

Decreased serum total cholesterol in E-selectin tolerized mice is of interest and further study is needed to characterize the mechanisms responsible for this reduction. It is however notable, that immune cells have been shown to directly influence plasma total cholesterol level [34] and that increased IL-10 expression reduces serum total cholesterol in ApoE**−/−** mice [35].

In the present study, intranasal delivery of E-selectin gave rise to E-selectin specific responses measureable by a reduction in Th1 mediated hypersensitivity, E-selectin specific cytokine production as well as E-selectin-specific IgG/IgG1 antibodies consistent with an immunosuppressive isotype. Intranasal delivery of E-selectin reduced DTH, an antigen-specific Th1 response, in C57BL/6 mice with efficacy similar to that previously observed in rats [24], [26]. No significant reductions in swelling/responses were observed in animals receiving the irrelevant antigen OVA, further emphasizing the importance of specificity for E-selectin. Cytokine and antibody responses were detectable in both regimens A and B, yet slightly greater proliferation and cytokine responses in regimen B may reflect an association between the robustness of responses and the greater time for responses to develop after tolerization has been completed (2 weeks in regimen A vs. 7 weeks in regimen B). Mucosal delivery of antigen can induce antigen specific regulatory or tolerogenic cells [16], [36], [37], [38], thus intranasal delivery of E-selectin in atherosclerosis plausibly involves the generation of E-selectin specific regulatory cells. We observed increased IL-10 and IL-2 production upon E-selectin stimulation in splenocytes from E-selectin treated mice. IL-10 was the primary effector cytokine produced in tolerized animals in response to E-selectin, and was also increased in splenocyte cultures of E-selectin tolerized animals exhibiting less ischemic brain injury [24]. IL-10 producing cells possess a regulatory capacity and studies have shown that IL-10 itself is anti-atherogenic [39], [40], [41]. While IL-2 is considered the prototypical autocrine cytokine and T cell mitogen [42], IL-2 is vital for the development, expansion, and function of Tregs [8], [43], [44].

E-selectin-specific IgG1 and total IgG were increased in mice receiving E-selectin with a relative paucity of IgG2a antibodies. However, the lesion sizes were not correlated with antibody titers (data not shown). The predominance of IgG1 over IgG2a antibodies is consistent with the immunomodulatory rather than proinflammatory nature of the response to E-selectin observed in lymphoid tissues, as it was reported that Th1 associated cytokines support the production of IgG2a antibodies while Th2 associated cytokines support the secretion of IgG1 antibodies in mice [45]. Quantitatively similar responses to human or mouse E-selectin by ELISA demonstrated the high degree of cross reactivity of antibodies generated in E-selectin tolerized animals and emphasizes the potential for responses against and efficacy in animals generating only the mouse form of E-selectin. The distinction between binding and neutralizing antibodies specific for E-selectin was beyond the scope of our study; however we cannot rule out any additional effects which may be antibody-mediated.

Our study demonstrated that E-selectin tolerization reduced gene expression of the chemoattractant cytokine CCL21, a key modulator of inflammation [46], [47] along with other lipid and inflammation-related molecules which were beyond the scope of this study. As well as being up-regulated in clinical and experimental atherosclerosis [48], CCL21 is also involved in neuroinflammation [49], [50], [51], suggesting how reductions in this chemokine secondary to intranasal E-selectin may also be beneficial in stroke.

Previous studies involving intranasal administration of E-selectin have implicated Tregs as contributors to reductions in vascular activation or associated immune responses. CD3 and Foxp3+ cells were negligible by IHC analysis, and mRNA expression of IL-10 or Foxp3 in the aorta was not different between treatment groups. This may be attributable to: 1) the timing of examination not being consistent with the presence of regulatory cells or 2) the location of antigen presentation and immunoregulation occurring outside the aorta. Notably, CCR7 – the ligand of CCL21 – is expressed at high levels on the majority of Foxp3+ Tregs [52], and reduced CCL21 expression in mice receiving E-selectin would foreseeably limit rather than enhance their recruitment at the site of disease.

Reduced serum active TGF-β in E-selectin tolerized mice may result from increased matrix deposition[53] or reduced production of active TGF-β. TGF-β has pro-atherogenic or anti-atherogenic effects in different stages of atherosclerosis [54], [55], [56]. As serum active TGF-β is correlated with coronary artery disease [57], [58], reduced serum active TGF-β in E-selectin tolerized mice may be consistent with benefit; however, an absolute or conclusive role for TGF-β in repair or pathogenesis remains unclear.

One may postulate deleterious effects associated with E-selectin administration, such as autoimmunity, interference with inflammatory cell trafficking and infiltration during tissue repair or infection resulting from immunosuppression. Upon challenge with influenza virus, mice receiving E-selectin showed similar resistance and survival to PBS-tolerized mice (John M. Hallenbeck, unpublished data, 2009). Because soluble E-selectin is critical in recruiting endothelial progenitor cells for repair processes [59], it is notable that the level of serum E-selectin is comparable between the treatment groups in our experiments limiting the potential for reduced progenitor recruitment. The effector profile of E-selectin specific cells as well as the antibody isotype is immunomodulatory rather than typical of proinflammatory or autoimmune-associated profiles. Moreover, IL-10 promotes regenerative healing in injured skin [60], recovery from spinal cord injury [61], and myocardial infarct recovery [62], thus its production in response to E-selectin likely contributes to protection rather than pathogenesis. The pattern of altered gene expression consistent with reduced inflammation and E-selectin tolerization found to augment brain remodeling after prolonged hypoperfusion in a model of post-ischemic adult neurogenesis [25] further suggest protective roles for E-selectin.

Additional studies are needed to investigate the long-term effect of E-selectin tolerization and to fully elucidate the role of E-selectin tolerization in atherosclerosis. The suppression of atherosclerosis, an important forerunner of acute ischemic cerebrovascular syndromes as well as diseases affecting other organs and vascular beds, provides further support for E-selectin tolerization as a promising strategy for prevention of stroke and other vascular diseases.
